# Malignant Giant Cell Tumor of Bone: A Clinicopathological Analysis of 15 Cases

**DOI:** 10.1155/humu/2709232

**Published:** 2026-11-06

**Authors:** Li Jiang, Jinmin Li, Yue Fan, Xiang Ling, Bin Mi, Benjamin Ka Seng Thong, Chaofu Wang, Binshen Ouyang

**Affiliations:** ^1^ Department of Pathology Ruijin Hospital Shanghai Jiao Tong University School of Medicine Shanghai China shsmu.edu.cn; ^2^ Department of Pathology The First People′s Hospital of Yulin Guangxi China; ^3^ Department of Pathology Guilin People′s Hospital Guangxi China; ^4^ Department of Pathology Pingyi County People′s Hospital Linyi China; ^5^ Shanghai Jiao Tong University School of Medicine Shanghai China shsmu.edu.cn

**Keywords:** bone tumors, clinical features, diagnosis, giant cell tumors

## Abstract

Malignant giant cell tumor of bone (MGCTB) refers to the presence of high‐grade sarcomatous components within a giant cell tumor of bone (GCTB). MGCTB is classified into primary (PMGCTB) and secondary (SMGCTB) forms. It is extremely rare and often misdiagnosed as other sarcomas, especially when typical GCTB morphology is absent, leading to diagnostic pitfalls. In this study, we analyze 15 cases of MGCTB, reviewing clinical data, pathological features, immunohistochemistry, molecular characteristics, and follow‐up outcomes. PMGCTB was found to be rarer than SMGCTB. Clinical symptoms predominantly include pain, mass detection, and restricted movement. MGCTB may lack GCTB components entirely, with malignant areas often presenting as high‐grade sarcomas, though low‐grade sarcomatous features can also be observed. Immunohistochemical markers such as H3.3G34W and H3G34W gene mutations exhibit high specificity and sensitivity for GCTB and MGCTB diagnosis. However, in cases with high dedifferentiation, few or absent mononuclear stromal cells may result in negative H3.3G34W expression and undetectable H3.3G34W gene mutations. The presence of high‐grade sarcomatous morphology in a giant cell‐rich tumor warrants thorough exclusion of PMGCTB. Additionally, misdiagnosis as SMGCTB can occur after denosumab treatment for GCTB. In conclusion, a comprehensive diagnostic approach combining patient age, tumor location, imaging, histology, immunohistochemistry, and molecular findings, with genetic testing when necessary, is essential for accurate diagnosis of GCTB and MGCTB. With a median follow‐up of 12 months (range, 1–120 months), the 1‐, 3‐, and 5‐year overall survival rates of the cohort were 76.9%, 76.9%, and 61.5%, respectively, and the median overall survival was not reached.

## 1. Introduction

Malignant giant cell tumor of bone (MGCTB) refers to the presence of high‐grade sarcomatous components within a giant cell tumor of bone (GCTB). MGCTB can be classified into two types: primary malignant giant cell tumor of bone (PMGCTB) and secondary malignant giant cell tumor of bone (SMGCTB). PMGCTB is characterized by the concurrent presence of high‐grade sarcomatous components within the GCTB, whereas SMGCTB occurs at the same site with high‐grade sarcoma following treatment of a pre‐existing GCTB (such as radiation therapy, chemotherapy, or surgery), with or without residual GCTB components.

MGCTB is exceedingly rare, accounting for approximately 4% of all GCTB cases. Among these, PMGCTB is even rarer, representing only 1.6%, whereas SMGCTB accounts for 2.4% of cases [1]. Because of the rarity of this tumor type and its potential for being misdiagnosed as other forms of sarcoma, particularly in the absence of classic GCTB features, there are significant diagnostic challenges. In this study, we analyzed 15 cases of MGCTB to further investigate their clinicopathological characteristics, highlight potential diagnostic pitfalls, and discuss the prognosis and progression of this rare entity.

## 2. Methods

### 2.1. Case Selection

This study was approved by the Institutional Review Board of the Department of Pathology at Ruijin Hospital, Shanghai Jiao Tong University School of Medicine (Shanghai, China). Fifteen MGCTB cases diagnosed between 2014 and 2024 were collected, including 12 in‐house and 3 consultation cases. Two experienced pathologists (C.W. and B.O.) independently reviewed the slides using a light microscope (BX43; Olympus, Tokyo, Japan). Clinical, imaging, and follow‐up data were obtained from the electronic medical system and through communication with referring institutions, patients, and families, with follow‐up completed in June 2024. The 12 in‐house MGCTB cases represented all consecutive MGCTB cases identified among all GCTB cases diagnosed at Ruijin Hospital during the same study period (2014–2024), retrieved from the institutional pathology archives with no additional selection criteria applied. The three consultation cases were referred from other institutions because of diagnostic difficulty; they were included because complete histological, immunohistochemical, and molecular workup was performed or verified at our institution, and because exclusion of these informative rare cases would further reduce the statistical power of an already small cohort. Potential referral bias introduced by the consultation cases is acknowledged as a limitation in Section 4.

### 2.2. Immunohistochemistry (IHC)

Tissue sections measuring 4 *μ*m were cut from formalin‐fixed, paraffin‐embedded tissue blocks and stained using commercially available antibodies, including H3.3G34W, SMA, P16, PGM1, CD68, SATB2 (ZSGB‐BIO, Beijing, China), P63, CD56, IDH1 (Bai‐Ling, Beijing, China), MDM2, CDK4, SOX10, CD34, S‐100, desmin, myogenin, MyoD1, Ki‐67 (Dako, Glostrup, Denmark), and AE1/AE3 (Dako, Carpinteria, California, United States). Clone names and working dilutions of the principal antibodies were as follows: H3.3G34W (clone RM263, 1:200; ZSGB‐BIO), P63 (clone 4A4, 1:100; Bai‐Ling), SATB2 (clone EP281, 1:200; ZSGB‐BIO), Ki‐67 (clone MIB‐1, 1:100; Dako), MDM2 (clone 1F2, 1:100; Dako), and CDK4 (clone DCS‐31, 1:100; Dako).

All specimens were formalin‐fixed, decalcified in 10% EDTA solution (pH 7.4) at room temperature, paraffin‐embedded, sectioned at 4 *μ*m, and H&E‐stained for microscopic examination. IHC was performed in‐house using the EnVision two‐step method, with heat‐induced epitope retrieval in EDTA buffer (pH 9.0) at 100°C for 20 min. CD68, CD34, and CD56 showed membrane positivity, PGM1, SMA, desmin, and AE1/AE3 were cytoplasmic, and H3.3G34W, P63, MDM2, CDK4, SATB2, S‐100, SOX10, myogenin, MyoD1, IDH‐1, and Ki‐67 were nuclear. P16 showed both cytoplasmic and nuclear positivity. Antibody binding was detected with the EnVision system (Dako), and results were interpreted by two pathologists (B.O. and C.W.). The same decalcified, paraffin‐embedded blocks were used for IHC and subsequent DNA extraction.

### 2.3. PCR and Sanger Sequencing Analysis

Primers were designed specifically for the detected pathogenic variant sites in the H3F3A gene. DNA was extracted from both the tumor and adjacent normal tissue using the QIAamp DNA FFPE Tissue Kit (Qiagen, United States). PCR amplification and sequencing were then performed according to the manufacturer′s instructions, using the extracted DNA as the template. DNA concentration was measured using the Qubit assay (BGI, China). The sequencing results were compared with the H3F3A reference gene sequence from a genomic database to identify potential mutations. Only H3F3A was analyzed; H3F3B was not included in the sequencing panel. PCR products were sequenced bidirectionally by Sanger sequencing, and the resulting chromatograms were compared with the H3F3A reference sequence (RefSeq NM_002107). For decalcified samples, DNA was extracted from the EDTA‐decalcified FFPE blocks using the same protocol, and PCR cycle numbers were increased when template quality was suboptimal.

## 3. Results

### 3.1. Clinical Characteristics

Among the 15 cases of MGCTB, 2 were PMGCTB and 13 were SMGCTB. The two PMGCTB cases involved males aged 47 and 69, who presented with pain or palpable masses with mild tenderness, both occurring in the distal femur. Of the 13 SMGCTB cases, five were females and eight were males, with ages ranging from 28 to 72 years (mean age: 52.1 years). Initial symptoms of GCTB included pain at the affected site (nine cases), fractures due to falls (two cases), palpable mass (one case), and detection during routine examination postimplantation of a bone plate (one case). Symptoms of SMGCTB included pain with or without swelling at the primary site (10 cases), detection during routine examination (one case), fever with prosthesis displacement (one case), and hemoptysis (one case).

The anatomical distribution of SMGCTB was as follows: proximal femur (five cases), distal femur (three cases), proximal tibia (two cases), distal tibia (one case), scapula (one case), and distal ulnar (one case). The latency period ranged from 2 to 36 years, with an average of 15.1 years. Prior to the diagnosis of secondary malignancy, one case had a history of radiotherapy, two cases had received denosumab treatment, and one case had both radiotherapy and denosumab treatment (Table 1).

**Table 1:** Clinicopathological features of malignant giant cell tumor of bone (MGCTB).

Case	Gender	Age (years)	Location	Max diameter (cm)	Treatment during latent period (1: radiation, 2: denosumab, 3: both, 4: none)	Latent period (years)	Pathological diagnosis	Immunophenotype	H3F3A gene mutation	Follow‐up (years) and outcome
1	Male	47	Distal femur	10	—	—	PMGCTB, malignant component: conventional osteosarcoma	P63‐, H3.3G34W+	Positive	3 years, recurrence, survival
2	Male	69	Distal femur	6	—	—	PMGCTB, malignant components: conventional osteosarcoma, chondrosarcoma	P63‐, H3.3G34W‐, SATB2 (partial+), D2‐40 (chondrocyte+)	Positive, IDH1/IDH2 negative	6 months, died of COVID‐19
3	Female	60	Proximal femur	30	1	7	SMGCTB, malignant component: conventional osteosarcoma	P63‐, H3.3G34W‐	Positive	10 years, lost to follow‐up
4	Female	43	Distal femur	8	4	7	SMGCTB, malignant component: conventional osteosarcoma	P63‐, H3.3G34W+	Positive	10 years, lost to follow‐up
5	Female	28	Proximal tibia	3	4	6	SMGCTB, malignant component: undifferentiated sarcoma	P63‐, H3.3G34W+	Positive	6 years, no recurrence, healthy
6	Female	42	Distal tibia	9	4	3	SMGCTB, malignant component: conventional osteosarcoma	P63 (some +), H3.3G34W+	Positive	9 years, lost to follow‐up
7	Female	72	Proximal tibia	7.3	4	29	SMGCTB, malignant component: undifferentiated sarcoma	P63‐, H3.3G34W‐	Positive	1 month, no recurrence, healthy
8	Male	57	Proximal femur	14	4	31	SMGCTB, malignant component: conventional osteosarcoma	P63 (partial+), H3.3G34W‐	Positive	4 years, widespread metastasis, death
9	Male	66	Scapular neck and body	14	4	18	SMGCTB, malignant component: low‐grade osteosarcoma	P63 (partial+), H3.3G34W+	Positive	1 year, recurrence, progression to high‐grade sarcoma, healthy
10	Male	39	Distal femur	7	2	3	SMGCTB, malignant component: conventional osteosarcoma	P63 (some +), H3.3G34W+	Positive	1 year, recurrence, survival
11	Male	64	Distal femur	10	2	27	SMGCTB, malignant component: conventional osteosarcoma	P63‐, H3.3G34W+	Positive	1 year, no recurrence, healthy
12	Male	44	Distal ulna	8	4	10	SMGCTB, malignant component: conventional osteosarcoma	P63 (partial+), H3.3G34W+	Positive	1 month, no recurrence, healthy
13	Male	65	Proximal femur	6	4	36	SMGCTB, malignant component: conventional osteosarcoma	P63 (some +), H3.3G34W‐	Negative	1 year, widespread metastasis, survival
14	Male	50	Proximal femur	3.7	3	2	SMGCTB, malignant component: undifferentiated sarcoma	P63 (few +), H3.3G34W‐	Negative	1 year, widespread metastasis, death
15	Male	48	Proximal femur	40	4	18	SMGCTB, malignant component: conventional osteosarcoma	P63 (partial+), H3.3G34W+	Negative	1 year, died of hemoptysis due to lung metastasis

Abbreviations: PMGCTB, primary malignant giant cell tumor of bone; SMGCTB, secondary malignant giant cell tumor of bone.

For the two PMGCTB cases, one died of COVID‐19 after 6 months, and one survived with the tumor. For the 13 SMGCTB cases, with follow‐up ranging from 1 month to 10 years, four cases showed no recurrence and remained healthy, three cases experienced recurrence or metastasis within a year but survived, three cases died from extensive metastasis, and three cases were lost to follow‐up. The median follow‐up duration for the entire cohort was 12 months (range, 1–120 months). Kaplan–Meier analysis of overall survival showed 1‐, 3‐, and 5‐year survival rates of 76.9%, 76.9%, and 61.5%, respectively.

### 3.2. Radiologic Characteristics

PMGCTB on CT shows discontinuous bone cortex, uneven density, multiple low and high‐density speckled shadows, and surrounding soft tissue masses (Figure 1A). MRI reveals interrupted cortical signals, patchy abnormal signals in the bone marrow cavity, and surrounding soft tissues (Figure 1B). SMGCTB on CTA displays bone destruction, surrounding soft tissue masses with uneven internal density, well‐defined or unclear boundaries with surrounding tissues, varying degrees of heterogeneous enhancement, and in some cases, multiple calcifications or cystic density shadows. MRI shows soft tissue masses with mixed signal intensities on T2W, low signal on T1W, and variable high, medium, or low signals on STIR (Figure 1C), with well‐defined or unclear boundaries with surrounding tissues.

**Figure 1:**
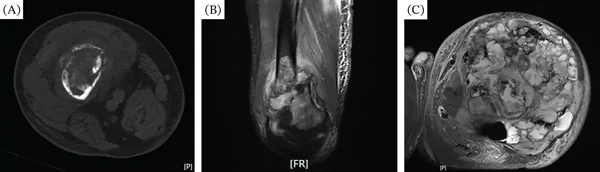
Radiologic characteristics of MGCTB. (A,B) A 69‐year‐old male with PMGCTB of the right distal femur. CT reveals cortical bone discontinuity, uneven density, multiple spotted low and high‐density areas, and a soft tissue mass. MRI shows interrupted cortical bone signal and abnormal signals in the bone marrow and surrounding soft tissue. (C) A 48‐year‐old male with a giant 40.0 × 16.0 × 16.0‐cm tumor in the left proximal femur. SMGCTB on MRI reveals a soft tissue mass with mixed equal and high signals on T2W, low signal on T1W, and variable signals on STIR. The boundary with surrounding soft tissue is clear.

The radiological features showed partial correspondence with histopathological findings (Table 2). In SMGCTB cases with osteosarcomatous components (Cases 3, 6, and 8), imaging typically demonstrated expansile lytic lesions with multiple coarse calcifications, which histologically corresponded to neoplastic osteoid formation—osteoid matrix directly produced by malignant osteoblastic cells. In cases with undifferentiated sarcoma components (Cases 5 and 7), imaging predominantly showed purely lytic destruction without significant calcification or ossification, as the sarcoma cells produced neither osteoid nor chondroid matrix. These correlations suggest that radiological features may partially reflect the histological differentiation of MGCTB; however, definitive diagnosis still relies on comprehensive histopathological and molecular evaluation.

**Table 2:** Imaging‐pathology correlation of malignant giant cell tumor of bone (MGCTB).

Case no.	Malignant component (pathology)	Key imaging features	Corresponding histopathological findings	Imaging‐pathology correlation
3	SMGCTB, malignant component: conventional osteosarcoma	Periprosthetic soft tissue mass with calcification; bone destruction	Malignant osteoid production around prosthesis; invasive growth into soft tissue	Periprosthetic mass with calcification corresponds to reactive/tumor bone formation; bone destruction indicates invasion
5	SMGCTB, malignant component: undifferentiated sarcoma	Purely lytic destruction; no calcification or ossification	Undifferentiated sarcoma cells; no osteoid or chondroid matrix production	Pure lytic pattern reflects absence of matrix‐producing differentia
6	SMGCTB, malignant component: conventional osteosarcoma	Lytic lesion with focal calcification; well‐defined margin (MRI)	Osteosarcomatous component with focal osteoid; pseudocapsule on gross; microscopic vascular invasion	Calcification corresponds to focal osteoid; well‐defined margin does not rule out microscopic invasion
7	SMGCTB, malignant component: undifferentiated sarcoma	Aggressive osteolytic destruction; soft tissue mass	Undifferentiated sarcoma; no osteoid production; extensive necrosis	Pure lytic destruction reflects undifferentiated morphology without matrix production
8	SMGCTB, malignant component: conventional osteosarcoma	Extensive bone destruction; multiorgan metastases	High‐grade osteosarcoma with osteoid production; metastatic deposits in multiple organs	Extensive destruction and metastases correspond to highly aggressive sarcomatous behavior

### 3.3. Macroscopic Characteristics

The tumors varied in size from 3.0 to 40.0 cm in diameter, with an average size of 11.7 cm. PMGCTB had maximum diameters of 6 and 10 cm, respectively, whereas SMGCTB had an average maximum diameter of 12.3 cm. Most cross‐sections appeared gray‐white or gray‐red, with some gray‐yellow areas. The texture varied from firm to soft, or with a fish‐flesh–like texture. Focal lesion could be hard and calcified, with hemorrhage, necrosis, or cystic changes. These characteristics can occur alone or simultaneously.

### 3.4. Histopathology, Immunophenotype, and Molecular Features

Among the two PMGCTB cases, one showed a clear demarcation between the GCTB area and the conventional osteosarcoma area, with coagulative necrosis. The other lacked obvious GCTB morphology, with malignant components partly as conventional osteosarcoma (Figure 2A) and partly as chondrosarcoma (Grade 2–3) (Figure 2B). Among the 13 SMGCTB cases, all showed secondary malignant transformation at the surgical site post‐GCTB surgery. The tumor cells infiltrated the surrounding tissue and underwent osteogenesis, producing bone matrix, resulting in conventional osteosarcoma in nine cases, undifferentiated sarcoma in three cases (Figure 2C), and low‐grade osteosarcoma in one case (Figure 2D). Additional features included hemorrhage and necrosis in five cases, aneurysmal bone cyst formation in one case, and intravascular tumor emboli in one case.

**Figure 2:**
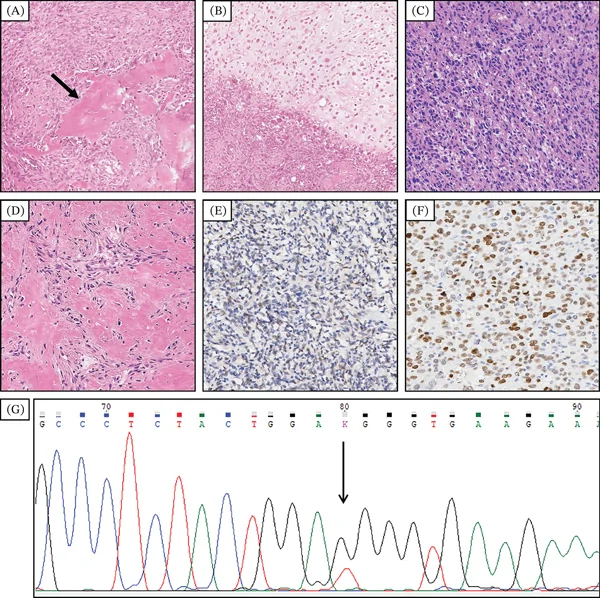
Histopathology, immunophenotype, and molecular features of MGCTB. (A,B) H&E slides of PMGCTB with conventional osteosarcoma (A, 10×, arrow indicating tumor cells forming bone) and chondrosarcoma (Grade 2–3), clearly demarcated (B, 10×). (C) H&E slide of SMGCTB with undifferentiated sarcoma, marked by significant atypia and pathological mitoses (10×). (D) H&E slide of SMGCTB with low‐grade osteosarcoma, featuring spindle‐shaped cells with mild atypia forming bone (10×). (E,F) P63 and H3.3G34W positive in the nuclei of SMGCTB tumor cells, respectively (10×). (G) The sequencing graph of the H3F3A gene mutation detected by PCR‐Sanger sequencing: A base G is mutated to base T, indicating the H3F3A gene G34W mutation.

P63 was expressed in 53.3% (8/15) of MGCTB cases: 0% in PMGCTB (0/2) and 61.5% in SMGCTB (8/13) (Figure 2E). H3G34W positivity was 60.0% (9/15) in MGCTB cases: 50% in PMGCTB (1/2) and 61.5% in SMGCTB (8/13) (Figure 2F). H3F3A gene G34W mutation was present in 80.0% (12/15) of MGCTB cases: 100% in PMGCTB (2/2) and 76.9% in SMGCTB (10/13). For PMGCTB, one case was P63‐negative, H3.3G34W‐positive, and had a positive H3F3A gene mutation test. The other case was negative for both P63 and H3.3G34W but had a positive H3F3A gene mutation test. IDH1/IDH2 gene tests were negative (Figure 2G). For SMGCTB, Cases 10, 11, and 13 were previously positive for H3.3G34W, P63, and H3F3A gene mutation during GCTB (Table 1).

## 4. Discussion

This study analyzes 15 cases of MGCTB, comprising 2 PMGCTB and 13 SMGCTB. We examined clinical presentation, imaging, pathology, molecular features, follow‐up, and prognosis. The transformation from GCTB to MGCTB often exceeds 3 years, with two cases exceeding 30 years. MGCTB shows a male predominance, and in rare cases, may lack typical GCTB morphology, leading to misdiagnosis. Malignant components are primarily high‐grade sarcomas, though low‐grade osteosarcoma and chondrosarcoma can also occur. Immunohistochemical detection of H3.3G34W and molecular analysis of H3F3A mutations demonstrate high specificity and sensitivity for diagnosing GCTB and MGCTB. In highly dedifferentiated MGCTB cases, reduced or absent mononuclear stromal cells can result in undetectable H3.3G34W. Very few MGCTB cases show mutations in non‐34W sites of the H3F3A gene, necessitating gene sequencing for accurate diagnosis. Literature reporting high‐grade sarcomas rich in giant cell components showing H3F3A mutations raises questions, as these cases may actually be PMGCTB lacking classic GCTB morphology. Although P63 is sensitive for diagnosing GCTB and MGCTB, our institutional data do not validate the proposed threshold of > 50% strong P63 positivity in tumor cells as a diagnostic criterion for GCTB. After denosumab treatment, GCTB may pose diagnostic challenges and be misidentified as SMGCTB.

MGCTB is a rare sarcoma originating from bone, first described by Stewart, with early researchers like Hutter and Dahlin distinguishing PMGCTB from SMGCTB [2–4]. MGCTB can occur in any bone, most commonly in the distal femur and proximal tibia. Among our 15 MGCTB cases, the distal and proximal femur each represented 33.3% (5/15), whereas the proximal tibia accounted for 13.3% (2/15). Local recurrence of GCTB typically occurs within 2–3 years [5, 6]. Cases transitioning from GCTB to SMGCTB after short‐term treatment should prompt further investigation into possible missed malignant components. SMGCTB usually appears after 3 years, with intervals of 3–20 years from the initial GCTB diagnosis, although cases extending beyond 40 years have been reported [6–8]. In our 13 SMGCTB cases, only one had a latency period of 2 years, whereas others ranged from 3 to 36 years. The male‐to‐female ratio in MGCTB is 1.28–3.25; our study found a ratio of 2.0 (10:5) [7, 8]. Typical symptoms include pain, swelling, and restricted motion. MGCTB lacks unique clinical or imaging features; x‐rays typically show eccentric lytic soapy changes at long bone metaphyses. CT reveals soft tissue masses with well‐defined lytic lesions, whereas MRI provides better detail of intramedullary and soft tissue extent [8].

PMGCTB almost always shows a mixture of conventional GCTB and high‐grade sarcoma areas, which can either sharply transition or gradually merge; however, there are reports of cases with no GCTB morphology at all [9]. GCTB is primarily composed of sheets of mononuclear stromal cells, mononuclear macrophages, and scattered uniformly distributed osteoclast‐like multinucleated giant cells. The stromal cell nuclei are round or oval, with abundant eosinophilic or amphophilic cytoplasm and indistinct cell borders. The nuclear morphology of the mononuclear stromal cells and the osteoclast‐like multinucleated giant cells is similar, and it is difficult to distinguish mononuclear stromal cells from mononuclear macrophages under H&E staining. The osteoclast‐like multinucleated giant cells are uniformly dispersed within the stroma, with the number of nuclei ranging from 10 to over 100. The high‐grade sarcoma component of MGCTB is most commonly conventional osteosarcoma, followed by fibrosarcoma and undifferentiated sarcoma [6, 8]. Sarcoma cells exhibit significant atypia and pleomorphism, with frequent pathological mitoses, and may be accompanied by hemorrhage, necrosis, and aneurysmal bone cyst formation. In our series of two PMGCTB cases, one case, after adequate sampling, did not show any conventional GCTB regions. The malignant components were conventional osteosarcoma and chondrosarcoma, and molecular testing revealed an H3F3A G34W mutation with no IDH1/IDH2 mutations, supporting the diagnosis of PMGCTB. SMGCTB is characterized by the appearance of a sarcoma at the primary site in patients with a previous history of GCTB, with or without GCTB morphological features. The sarcoma components in SMGCTB are similar to those in PMGCTB, most commonly conventional osteosarcoma, followed by fibrosarcoma and undifferentiated sarcoma. Among our SMGCTB cases, the malignant components included conventional osteosarcoma in nine cases, undifferentiated sarcoma in three cases, and low‐grade osteosarcoma in one case. Some studies have shown a certain correlation between radiotherapy and SMGCTB, especially in cases where GCTB recurs several years after radiotherapy, raising the possibility of MGCTB [10, 11], although the pathogenic mechanism remains unclear and has not yet been widely accepted.

Reports have indicated that the malignant component of MGCTB can also exhibit low‐grade central osteosarcoma‐like morphology, reflecting histological variations of the tumor [9]. In this cohort of SMGCTB cases, a malignant component with low‐grade sarcoma morphology was observed in one instance. The patient was a 66‐year‐old male who was hospitalized due to “swelling in the left shoulder for more than three months.” He had a history of “giant cell tumor of the left scapula” for over 17 years. The tumor within and around the scapula measured 14.0 × 10.0 × 8.0 cm and was multinodular with a fused appearance. Under H&E morphology, spindle‐shaped tumor cells exhibited mild atypia, and no clear osteoblasts were observed; the tumor cells directly produced osteoid matrix (Figure 3A). Even after extensive sampling, high‐grade sarcoma components were not detected. Immunohistochemical analysis showed that tumor cells expressed H3.3G34W (Figure 3B), CD56 (Figure 3C), and SATB2, with low Ki‐67 expression (Figure 3D). Molecular testing identified the H3F3A gene G34W mutation (Figure 3E). Given the previous diagnosis of GCTB, the diagnosis of SMGCTB was established, with the malignant component being low‐grade osteosarcoma. This indicates that the malignant components of MGCTB are not restricted to high‐grade sarcomas, and it is believed that low‐grade sarcoma morphology in MGCTB will be discovered and reported more frequently in the future. After 10 months of follow‐up, the patient experienced a recurrence of the lesion, with the malignant component progressing to high‐grade sarcoma (undifferentiated sarcoma), whereas the H3F3A gene G34W mutation was still detected.

**Figure 3:**
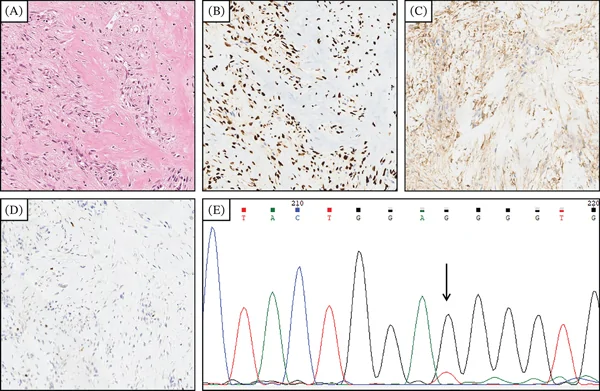
Morphology, immunophenotype, and molecular features of low‐grade central osteosarcoma in MGCTB. (A) Spindle‐shaped tumor cells with mild atypia and no clear osteoblasts observed. Tumor cells directly produce osteoid matrix (10×). (B–D) (10×) Immunohistochemical staining, with tumor cells expressing H3.3G34W (10×) and CD56 (10×), and low Ki‐67 expression. (E) PCR‐Sanger sequencing result for detecting the H3F3A gene mutation: a base substitution from C to A, identifying the H3F3A gene G34W mutation.

The imaging heterogeneity of SMGCTB following malignant transformation is closely related to its histopathological diversity. The tumor is composed of three basic components—mononuclear stromal cells, osteoclast‐like multinucleated giant cells, and tumor osteoid/woven bone—in varying proportions. When osteoid production is abundant and well‐mineralized, imaging reveals dense calcification or bone‐like density; when the tumor stroma contains numerous thin‐walled vascular sinusoids prone to recurrent hemorrhage and cystic change, cystic density or hyperintensity on T2WI emerges, with severe cases presenting as secondary aneurysmal bone cyst‐like changes. Thus, the “mixed” appearance on imaging is essentially a direct macroscopic reflection of the heterogeneous spatial distribution of these three components. In addition, the imaging findings of extraosseous extension and distant metastases are consistent with the pathological progression toward high‐grade sarcoma. Understanding this imaging‐pathology correlation may assist surgeons in preliminarily assessing the biological composition of the tumor preoperatively, thereby optimizing surgical strategy and guiding postoperative surveillance.

P63 is a member of the transcription factor family and is positively expressed in the mononuclear stromal cells of GCTB, whereas it is not expressed in mononuclear macrophages or osteoclast‐like giant cells. In MGCTB, the intensity and quantity of P63 positive expression negatively correlate with the degree of malignant transformation of true mononuclear stromal cells. P63 has good sensitivity for diagnosing GCTB and MGCTB but lacks specificity; it is often expressed in other giant cell‐rich lesions, such as aneurysmal bone cysts, chondromyxoid fibromas, and osteosarcomas. In 15 cases of MGCTB, the expression rate of P63 was 53.3% (8/15), with a 0% expression rate in PMGCTB (0/2) and a 61.5% expression rate in SMGCTB (8/13), presenting as focal, partial, or mottled positivity. This may be due to a high degree of tumor dedifferentiation and a reduced number of residual mononuclear stromal cells affecting P63 expression, which can even be completely absent. Some literature reports have shown strong positive expression of P63 in mononuclear stromal cells in GCTB [12], with the percentage of positive tumor cells ranging from 50.5% to 71%. Although there is expression in other giant cell‐rich lesions, the staining is weak to moderate, with positive tumor cells comprising less than 50%. A threshold of more than 50% strong positive tumor cells can be used to diagnose GCTB. However, Shooshtarizadeh et al. [13] hold a different view, reporting that strong staining of tumor cells was found in 35.3% of chondrosarcomas and 7.1% of osteosarcomas, with diffuse expression of P63 observed in 23.5% of chondrosarcomas and 14.3% of osteosarcomas. In this group of 15 MGCTB cases, three previously diagnosed GCTB cases tested positive for P63, with a sensitivity of 100% (3/3). However, only one case had strong positivity in mononuclear stromal cells, with a percentage greater than 50%. The other two cases showed weak to moderate staining, with percentages less than 50%. Taken together, our institutional experience does not validate the > 50% strong‐positivity threshold: Only one of the three previously diagnosed GCTB cases in this cohort met this criterion, whereas the other two showed weak‐to‐moderate staining in fewer than 50% of tumor cells. In practice, P63 should be regarded as a sensitive but nonspecific adjunctive marker for GCTB and MGCTB, the > 50% threshold should not be used as a stand‐alone diagnostic criterion, and confirmation with H3.3G34W IHC and/or H3F3A mutation analysis is recommended when P63 findings are equivocal.

H3G34W is an extremely specific and sensitive antibody for diagnosing GCTB and MGCTB and is rarely expressed in diseases rich in giant cell morphology [9, 14–16], such as osteosarcomas, aneurysmal bone cysts, nonossifying fibromas, chondrosarcomas, giant cell tumors of the tendon sheath, and brown tumors. Up to 96% of GCTB cases have H3F3A gene mutations. Although mutations can also be detected in MGCTB, they have no significant value in distinguishing between benign and malignant tumors. H3G34W is expressed in the nuclei of mononuclear stromal cells with H3F3A gene mutations but expression is limited by sample quality and tumor cell numbers, especially in PMGCTB, where H3F3A mutations may be retained or absent [17]. Moreover, it cannot detect other types like G35L, G35M, G35R, G35V, and wild types, so a negative H3G34W cannot exclude the diagnosis of GCTB or MGCTB. When clinical, histological morphology, and imaging all raise suspicion of GCTB, the absence of H3G34W expression should prompt additional testing for H3F3A gene mutations, and DNA sequencing analysis may be needed for further diagnostic assistance. In this study, the positive rate of H3G34W was 60.0% (9/15) among 15 MGCTB cases, whereas the mutation rate of the H3F3A gene G34W was 80.0% (12/15). Among the six H3G34W negative MGCTB cases, four were confirmed to have H3F3A mutations by genetic testing. It is considered that the negative results may be due to decalcification processing of the tissue or long storage duration of the specimens (5.5 years), leading to decreased or even degraded gene and protein expression. One case showed both H3G34W and H3F3A gene mutations as negative, but H3F3A mutations were detected in previous GCTB. It is considered that during malignant transformation, tumor cells may lose their original differentiation direction, and with fewer or absent residual mononuclear stromal cells, the H3.3G34W gene could not be detected. Yoshida et al. [17] also reported on this situation, suggesting that the deletion of H3F3A alleles may be a reason for this special phenomenon. Another MGCTB case showed both H3G34W and H3F3A gene 34W site mutations as negative. Possible reasons include mutations at other sites of H3F3A (non‐G34 sites) or further molecular changes during malignant transformation, rendering detection impossible. Alternatively, it could be due to a heterozygous deletion of the H3F3A allele (while preserving the wild‐type allele) [17]. Amary et al. [9] conducted a retrospective study revealing 13 cases of primary malignant bone tumors carrying G34 mutations, among which 11 had G34W mutations and 2 had G34R mutations. The expression rate of H3G34W was 2.85% (11/385). However, subsequent analysis showed that these high‐grade sarcomas all occurred in common sites of GCTB and MGCTB (proximal and distal femur) and were high‐grade sarcomas rich in giant cells, without completely excluding PMGCTB. Yamamoto et al. [18] also observed a case of diagnosed primary undifferentiated pleomorphic sarcoma (UPS) that showed H3.3 G34W expression and H3F3A mutations. However, the authors speculated that this case might be PMGCTB without classic GCTB features, accompanied by excessive growth of sarcoma components, or it could be an ordinary UPS unrelated to GCTB that indeed had H3F3A mutations. Considering that the molecular detection of H3F3A mutations is a highly specific and sensitive indicator for GCTB and MGCTB and is rarely expressed in other malignant tumors [14–16], the fifth edition of the WHO classification of bone tumors also supports the diagnosis of MGCTB if H3F3A gene mutations are detected in primary malignant bone tumors of the epiphysis. In summary, the authors believe that it is highly likely to be PMGCTB lacking classic GCTB morphology.

It is noteworthy that although the H3F3A G34W mutation is highly sensitive and specific for GCTB, it has been occasionally reported in malignant tumors such as giant cell‐rich osteosarcoma [9, 19]. Therefore, the presence of this molecular marker must be interpreted in conjunction with histological features (particularly the exclusion of neoplastic osteoid formation) and clinicoradiological findings and should not be used in isolation as the sole diagnostic criterion for MGCTB.

The transformation of GCTB into a malignant tumor can take years or decades, but the molecular changes during this process remain unclear. H3F3A mutations have been detected in the stromal cells of GCTB and MGCTB. One study reported that 96% of GCTB cases harbor histone H3F3A (H3.3) gene mutations, involving glycine 34 substitutions, with the majority represented by p.Gly34Trp (p.G34W), and a few cases having G34L, V, and M variants. Rare H3.3 variants such as G34L, V, and M are most commonly found in small bones of the hands and feet, patella, and axial skeleton. Oda et al. [20] reported that TP53 mutations may be associated with the malignant progression of GCTB. Fittall et al. [21] observed that, in addition to H3F3A or H3F3B mutations, most MGCTB cases exhibit features associated with TERT promoter mutations, suggesting that telomere dysfunction may play a role in the malignant transformation of GCTB. Biallelic loss of the histone lysine demethylases KDM4B or KDM6A is also a nonrecurrent event.

The mononuclear stromal cells in GCTB produce several cytokines and mediators, including the receptor activator of the nuclear factor *κ*‐B ligand (RANK‐RANKL) system, which recruits and activates osteoclast‐like giant cells [6]. Denosumab, a RANKL inhibitor, blocks bone resorption by depleting osteoclasts through the RANKL pathway and is often used in the clinical treatment of GCTB. However, true mononuclear stromal cells are not suppressed, and H3.3 G34W can mark surviving tumor cells [22]. Whether denosumab promotes the transformation of GCTB into MGCTB remains inconclusive [23], with most reports being case studies. Among the 13 SMGCTB cases in this study, three were diagnosed after denosumab treatment, with one case developing osteosarcoma and two developing undifferentiated sarcoma. One case was diagnosed with SMGCTB after 4 months of denosumab treatment; another case was diagnosed with SMGCTB 2.5 months after denosumab treatment following a recurrence indicated by MRI 2 years after surgery on the primary GCTB lesion, raising questions about whether malignancy existed before denosumab treatment or whether the transformation occurred within the 2.5‐month treatment period. A third case was diagnosed with SMGCTB 14 months after denosumab treatment, with lung metastases confirmed by biopsy before denosumab treatment, although malignant components in the metastasis were not confirmed. Liu et al. [8] reported a biopsy false‐negative rate of up to 87.5%, and this patient had a history of radiation therapy, which itself can contribute to malignant transformation of GCTB [1]. Rutkowski et al. [24] reported two cases of malignant transformation during denosumab treatment, suggesting that these were PMGCTB, but the malignant tissue component was not sampled during biopsy. Notably, denosumab treatment of GCTB can inhibit the RANK‐RANKL system, leading to a reduction or disappearance of osteoclast‐like giant cells, with spindle cell proliferation, atypical morphology, and reactive new bone formation, which could be misdiagnosed as SMGCTB [25]. However, the latter typically shows tumor shrinkage on imaging, with no bone matrix or new cortical bone formation on CT. Histologically, there is no significant atypia, mitotic figures are rare or absent, and osteoblasts are found around bone tissue with a reactive background. Ki‐67 indices typically range between 3% and 8% [26]. Therefore, more clinical data are needed to determine whether denosumab promotes malignant transformation of GCTB, as indicated by the results of this study.

We propose a diagnostic algorithm for SMGCTB and its differential diagnoses, as presented in Figure 4. The workflow begins with histomorphological identification of a giant cell‐rich bone lesion. H3.3G34W IHC serves as the primary screening tool: Diffuse nuclear positivity in mononuclear stromal cells (> 50% of tumor cells) supports a GCTB origin, with confirmatory H3F3A gene mutation testing recommended for equivocal cases. SATB2 positivity indicates osteoblastic differentiation, whereas MDM2 and CDK4 are employed to exclude low‐grade central osteosarcoma (diffuse positivity prompting FISH confirmation), and PGM1/CD68 highlights multinucleated giant cells. In H3.3G34W‐negative cases, other giant cell‐rich entities should be considered, including chondroblastoma (H3F3B K36M mutation), aneurysmal bone cyst (USP6 rearrangement), and giant cell‐rich osteosarcoma (SATB2 positivity with osteoid production). Molecular testing for the H3F3A G34W mutation was performed in all cases, with positivity confirming GCTB origin and negativity in metastatic lesions suggesting clonal evolution. Once GCTB is confirmed, the distinction between SMGCTB and PMGCTB relies on clinical history (prior GCTB at the same site) and latency period (typically > 2–3 years for SMGCTB, per WHO criteria). A Ki‐67 proliferation index ≥ 10% supports malignant transformation; this threshold was adopted because denosumab‐treated conventional GCTB without malignant transformation typically shows Ki‐67 indices of 3%–8% [26], and because a Ki‐67 index of ≥ 10% is commonly used to support malignancy in bone sarcomas. Finally, the malignant component is subtyped based on the presence or absence of osteoid production, categorizing tumors as secondary osteosarcoma or malignant GCTB with other sarcoma subtypes. However, in rare instances, loss of G34W immunoreactivity may occur following malignant transformation; therefore, a definitive diagnosis ultimately relies on the comprehensive integration of all available clinical data, radiological findings, histopathological features, immunohistochemical markers, and molecular testing results. This algorithm integrates morphological, immunophenotypic, and molecular data to provide a practical framework for accurate diagnosis.

**Figure 4:**
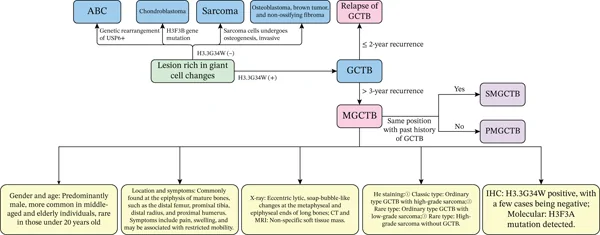
Proposed diagnostic flow for MGCTB. Step 1 (history/imaging): Prior GCTB > 5 years; aggressive features suggest transformation. Step 2 (histopathology): Sarcomatous components with/without adjacent GCTB areas. Step 3 (IHC): H3.3G34W (+) confirms GCTB origin; SATB2 (+) osteoblastic differentiation; MDM2/CDK4 (+) → FISH to exclude low‐grade central osteosarcoma; Ki‐67 ≥ 10% supports malignant transformation. Step 4 (molecular): H3F3A G34W mutation (+) confirms GCTB; MDM2 FISH amplification favors low‐grade central osteosarcoma. Step 5 (integrated diagnosis): Combine all clinical, radiological, histopathological, IHC, and molecular data. Abbreviations: GCTB, giant cell tumor of bone; MGCTB, malignancy in giant cell tumor of bone; PMGCTB, primary malignancy in giant cell tumor of bone; SMGCTB, secondary malignancy in giant cell tumor of bone; ABC, aneurysmal bone cyst; IHC, immunohistochemistry.

In cases of H3F3A‐mutant high‐grade sarcomas lacking GCTB morphology, we suggest the following approach to distinguish PMGCTB from incidental H3F3A‐mutant sarcomas. Clinical clues favoring PMGCTB include age 20–50 years, epiphyseal/metaphyseal location (long bones, knee region), and prior GCTB history or latency > 2–3 years. Histologically, thorough sampling (≥ 1 section/cm tumor) and deeper sectioning may uncover focal residual GCTB areas or an abrupt transition from GCTB to sarcoma; if absent, incidental mutation sarcoma should be considered. Immunohistochemically, diffuse H3.3G34W positivity in mononuclear cells and P63 expression support GCTB origin, but molecular evidence alone (H3F3A mutation) is insufficient for PMGCTB diagnosis without morphological confirmation. Application of these criteria may facilitate accurate diagnosis and appropriate clinical decision‐making in this challenging scenario.

Among two PMGCTB cases, one died from COVID‐19, and the other survived with the tumor. Among 13 SMGCTB cases, three patients died, three were lost to follow‐up (presumed deceased), three survived with the tumor, and four were healthy. There is no consensus on the best treatment for MGCTB, with surgery remaining the mainstay of treatment, although insufficient surgical margins are associated with a high recurrence rate [8]. Tahir et al. [6] noted that larger tumor size, older age, and the presence of metastases are associated with a worse prognosis in MGCTB, with distant metastases being the primary cause of mortality, consistent with the findings in this study. Because of the limited number of PMGCTB cases in this cohort, a formal prognostic comparison between primary and secondary malignant GCTB could not be performed. Future multicenter studies with larger sample sizes are needed to address this question. To provide a comprehensive overview of the follow‐up data and to address their fragmented presentation, we compiled a detailed case‐level summary table (Table S1) linking each patient′s malignant component, treatment history, latency period, and outcome. Analysis of this consolidated dataset revealed that among the 10 SMGCTB cases with available follow‐up, three patients died of disease (Cases 8, 14, and 15), all of whom developed widespread or pulmonary metastases. Notably, Case 15, with an 18‐year latency period and a giant 40‐cm proximal femoral tumor, exemplifies the destructive potential of late malignant transformation. The four patients who remained disease‐free (Cases 5, 7, 11, and 12) had undergone surgical resection with curative intent, with or without adjuvant chemotherapy, whereas those with incomplete resection or palliative treatment alone had poorer prognoses. These patterns suggest that metastatic status at malignant transformation and completeness of surgical resection, rather than latency period alone, may be the key determinants of survival in secondary malignant GCTB. Consistent with these patterns, Kaplan–Meier analysis of the whole cohort yielded 1‐, 3‐, and 5‐year overall survival rates of 76.9%, 76.9%, and 61.5%, respectively, with the median overall survival not reached (median follow‐up, 12 months; range, 1–120 months; Figure 5), although the small sample size precludes definitive prognostic conclusions.

**Figure 5:**
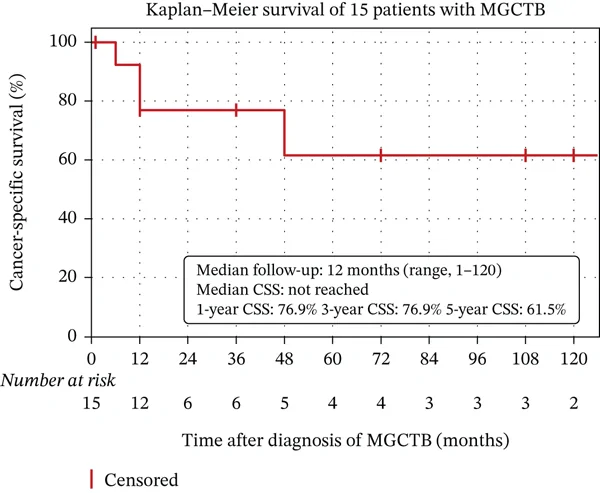
Kaplan–Meier overall survival curve of the 15 patients with MGCTB. Tick marks indicate censored observations (patients alive or lost to follow‐up at the last contact). The number of patients at risk at each time point is shown below the curve.

In our cohort, three SMGCTB cases were diagnosed following denosumab therapy. Only one of these (interval: 2.5 months) had a pretreatment biopsy confirming conventional GCTB without malignancy. Given the extremely short interval, we interpret this case as denosumab unmasking a pre‐existing malignant clone rather than inducing de novo transformation, as (1) 2.5 months is likely insufficient for denosumab to drive de novo malignant transformation; (2) H3F3A G34W mutation was present in the sarcomatous component, supporting a common clonal origin; and (3) sampling bias in GCTB biopsies is a well‐recognized limitation. In the other two cases without pretreatment pathological confirmation, we cannot definitively exclude either possibility. Overall, we caution that denosumab may accelerate the clinical manifestation of pre‐existing malignant clones by depleting benign giant cells and altering the histological appearance, rather than directly causing de novo malignant transformation. However, larger prospective studies are needed to confirm this interpretation. We acknowledge an important limitation of this interpretation: Predenosumab histological images and detailed morphological descriptions of the malignant components were not available for all three cases, and in the absence of such morphological evidence, the hypothesis that denosumab unmasks a pre‐existing malignant clone remains speculative. We therefore caution against overinterpretation and present this hypothesis strictly as one plausible explanation requiring validation in future studies with complete pretreatment and posttreatment histological sampling.

## 5. Conclusion

In MGCTB, GCTB components may be completely absent, with most malignant regions presenting as high‐grade sarcomas, although low‐grade sarcoma morphology can also occur. The immunohistochemical markers H3.3G34W and H3G34W, along with molecular detection, offer high specificity and sensitivity for diagnosing GCTB and MGCTB. However, in cases of high dedifferentiation in MGCTB, where mononuclear stromal cells are scarce or absent, H3.3G34W may yield negative results, making its gene undetectable. Our data do not support the previously proposed threshold of > 50% strong P63 positivity in tumor cells as a diagnostic criterion for GCTB, and P63 should instead be interpreted together with H3.3G34W IHC and H3F3A molecular findings. Given the age and common locations of MGCTB, if tumor cells exhibit high‐grade sarcoma morphology in a giant cell‐rich background, it is essential to rule out PMGCTB before considering other sarcomas. After denosumab treatment for GCTB, diagnostic pitfalls may lead to misdiagnosis as SMGCTB. Therefore, a comprehensive analysis that incorporates patient age, tumor location, imaging, histology, IHC, and molecular results is crucial for accurately diagnosing GCTB and MGCTB, with genetic testing employed as needed.

## Author Contributions

All authors contributed equally in the writing of this paper. Li Jiang, Jinmin Li, and Yue Fan contributed equally as first authors.

## Funding

No funding was received for this manuscript.

## Conflicts of Interest

The authors declare no conflicts of interest.

## Supporting information


**Supporting Information** Additional supporting information can be found online in the Supporting Information section. Table S1 summarizes the clinicopathological characteristics, treatment history, and clinical outcomes of 15 patients with malignant giant cell tumor of bone (MGCTB). It details the malignant component (histological subtype), treatment for primary GCTB and malignant transformation, and the outcome and follow‐up for each case. Cases 1 and 2 were diagnosed as primary malignant giant cell tumor of bone (PMGCTB) without prior GCTB history, whereas the remaining cases were secondary malignant giant cell tumor of bone (SMGCTB) arising from pre‐existing GCTB. These data provide detailed supporting clinical information for the study cohort.

## Data Availability

The data that support the findings of this study are available on request from the corresponding authors. The data are not publicly available due to privacy or ethical restrictions.
