# Supplementary material for: Malignant Giant Cell Tumor of Bone: A Clinicopathological Analysis of 15 Cases
Source: Hum Mutat. 2026 Nov 6;2026:2709232. doi: 10.1155/humu/2709232 (PMC13743633; doi:10.1155/humu/2709232)
Supplement: Supplementary file 1 [file HUMU-2026-2709232-s001.doc]

Supplementary Table S1. Clinicopathological Characteristics, Treatment History, and Clinical Outcomes of 15 Patients with Malignant Giant Cell Tumor of Bone (MGCTB).

| **Case No.** | **Malignant Component (Pathology)** | **Treatment of GCTB** | **Treatment for Malignant Transformation** | **Outcome & Follow-up** |
| --- | --- | --- | --- | --- |
| 1 | primary malignant giant cell tumor of bone | curettage + Bone cement filling | knee replacement + chemotherapy | 3 years, recurrence, survival |
| 2 | primary malignant giant cell tumor of bone, secondary osteosarcoma and chondrosarcoma | tumor resection | tumor resection + prosthetic replacement+hemoradiotherapy | 6 months, died of COVID-19 |
| 3 | Secondary osteosarcoma (periprosthetic) | curettage ; recurrence → arthroplasty + brachytherapy | hemipelvectomy | 10 years, lost to follow-up |
| 4 | Secondary osteosarcoma | curettage; recurrence → segmental resection + prosthesis | lesion debridement + Total femoral replacement | 10 years, lost to follow-up |
| 5 | Poorly differentiated sarcoma with epithelial differentiation | curettage + bone cement filling | knee arthroplasty | 6 years, no recurrence, healthy |
| 6 | Secondary osteosarcoma | curettage + bone cement filling | segmental resection + PORT implantation +chemotherapy | 9 years, lost to follow-up |
| 7 | secondary malignant GCTB, Secondary osteosarcoma (undifferentiated sarcoma) | tumor resection | tumor resection+prosthesis implantation | 1 month, no recurrence, healthy |
| 8 | secondary malignant GCTB, Secondary osteosarcoma | tumor resection ; recurrence → tumor resection + prosthetic replacement | chemotherapy | 4 years, widespread metastasis, death |
| 9 | secondary malignant GCTB, low-grade osteosarcomatous component | curettage + bone cement | scapulothoracic disarticulation + chemotherapy | 1 year, recurrence, progression to high-grade sarcoma, healthy |
| 10 | Secondary osteosarcoma (conventional type) | curettage + bone cement; recurrence → internal fixation+ denosumab | radiotherapy + chemotherapy | 1 year, recurrence, survival |
| 11 | secondary osteosarcoma (conventional type) | curettage ; Recurrence → arthroplasty + denosumab | amputation + chemotherapy | 1 year, no recurrence, healthy |
| 12 | Secondary osteosarcoma (conventional type) | curettage + Bone cement filling | scapulothoracic disarticulation + chemotherapy | 1 month, no recurrence, healthy |
| 13 | malignant GCTB (lung metastasis) | tumor curettage ; recurrence → joint replacement | denosumab + chemoradiotherapy | 1 year, widespread metastasis, survival |
| 14 | spindle cell component (lung metastasis) | tumor resection + prosthesis | radiotherapy + Chemotherapy | 1 year, widespread metastasis, death |
| 15 | secondary malignant GCTB, Secondary osteosarcoma | curettage + bone cement ; fracture → hip arthroplasty | chemoradiotherapy + hemipelvectomy + amputation | 1 year, died of hemoptysis due to lung metastasis |

This table summarizes the malignant component (histological subtype), treatment history for both primary GCTB and malignant transformation, and clinical outcomes with follow-up status for each case. Cases 1 and 2 were diagnosed as primary MGCTB (PMGCTB) without prior GCTB history; all other cases were secondary MGCTB (SMGCTB) arising from pre-existing GCTB.

Abbreviations: GCTB, giant cell tumor of bone; MGCTB, malignant giant cell tumor of bone; PORT, postoperative radiotherapy; COVID-19, coronavirus disease 2019.
